# Prevalence of migraine in Iran: a systematic review and meta-analysis

**DOI:** 10.1186/s12883-023-03215-5

**Published:** 2023-04-27

**Authors:** Payam Mohammadi, Mahbod Khodamorovati, Kamran Vafaee, Mahvan Hemmati, Niloufar Darvishi, Hooman Ghasemi

**Affiliations:** 1 Department of Neurology, School of Medicine, Kermanshah University of Medical Sciences, Kermanshah, Iran; 2 Clinical Research Development Center, Imam Reza Hospital, Kermanshah University of Medical Sciences, Kermanshah, Iran; 3 Medical Department, Georgia University of Medical Sciences, Tbilisi, Georgia; 4 Nursing Department, Nursing and Midwifery School, Kermanshah University of Medical Sciences, Kermanshah, Iran; 5 Student Research Committee, Kermanshah University of Medical Sciences, Kermanshah, Iran; 6 Department of Psychiatric Nursing, Faculty of Nursing School, Tehran Medical Sciences, Islamic Azad University Science and Research Branch, Tehran, Iran

## Abstract

**Background:**

Migraine headaches affect all ages, from childhood to old age. Migraine attacks cause significant changes in the living conditions of the sick person, including a decrease in personal, social, and occupational performance. This study was conducted to determine the prevalence of migraine in Iran through a systematic review and meta-analysis.

**Method:**

In this systematic review and meta-analysis study, the studies associated with the prevalence of migraine using the keywords: migraine, prevalence, and Iran its equivalents in international databases PubMed, Web of Science, Scopus, Science direct, and Iranian internal information databases, including SID and MagIran, was searched without limit until November 2022. Comprehensive Meta-Analysis software (Version 2) was used to analyze the data. Due to the high number of studies reviewed in this systematic review, the Begg and Mazumdar test was used at a significance level of 0.1, and the corresponding Funnel plot was used to check publication bias. Also, the I2 test was used to check the heterogeneity in this study.

**Results:**

22 records were included in the final analysis. The prevalence of migraine in the general population of Iran was 15.1% (confidence interval 95%: 10.7–20.9), and in this population, the prevalence of migraine was higher in women than in men. The prevalence of migraine based on The International Classification of Headache Disorders (ICHD) 2 criteria was reportedly 16.4% (95% CI: 10.8–24.1), and with ICHD3 criteria, this value was reported as 17.1% (95% CI: 7.7–33.6). Based on a survey of 4571 children, the prevalence of migraine was reported to be 5.2% (95% CI: 1.3–18.7). Also, the prevalence of migraine in adolescents was calculated based on eight studies (n = 8820). Accordingly, 11.2% (95% CI: 5.8–20.4) of adolescents have migraines. Meanwhile, the prevalence of migraine in boys was 8.2% (95% CI: 4.8–13.7), and in girls was 8% (95% CI: 6.2–12.7).

**Conclusion:**

As a result, the prevalence of migraine in Iran, based on population-based studies, was reported as 15.1%. The result showed a higher prevalence of migraine in the general population than in children and adolescents. It was also found that the prevalence of migraine in women is higher than in men.

**Supplementary Information:**

The online version contains supplementary material available at 10.1186/s12883-023-03215-5.

## Introduction

Migraine is a debilitating and common disease affecting millions worldwide [1]. The Headache Classification Committee of the International Headache Society (IHS) defines *migraine* as recurrent episodes of headache often accompanied by nausea, vomiting, photophobia, and phonophobia [2]. According to the results obtained from global burden disease 2019, migraine is the second cause of disability in the general population and the first disabling factor in women under 50 [3] in such a way that migraine attacks cause significant changes in the patient’s living conditions, including a decrease in personal, social and occupational performance [4].

Migraine prevalence varies in different geographical areas. Some studies have shown that the prevalence of migraine in Asian countries is lower than in other countries [5]. In general, the prevalence of migraine is reported between 2.6% and 21.7%, with an average of 12% [2]. The National Health Survey (NHIS) results in the United States showed that 15.3% of Americans, including 9.7% of men and 20.7% of women, reported migraines and severe headaches [6]. The review of epidemiological studies in European countries showed that the prevalence of migraine among adults is between 8 and 17.6%, while it is between 5.2 and 9.1% among children and adolescents[7]. In Iran, the prevalence of migraine in adults has varied from 5.4–41.6% [8, 9] and in children and adolescents from 4.8 to 28.1% in various studies [10, 11]. Based on the previous meta-analysis in Iran in 2016, the prevalence of migraine in Iran was reported as 14% [12].

Understanding prevalence as a function of age and sex informs how likely migraine might be in a particular patient. Also, discernment of how migraine manifests in different populations can improve the diagnosis of people with unusual presentations. On the other hand, a systematic review and meta-analysis study in 2016 investigated the prevalence of migraine in Iran. Based on this, the present study was conducted to update the results of the mentioned research and estimate the prevalence of migraine in Iran.

## Method

This study aimed to estimate the prevalence of migraine based on population-based studies in Iran and the prevalence of migraine in Iranian schoolchildren. Also, subgroup analyses were performed based on diagnostic criteria, gender, and age.

In this systematic review and meta-analysis, the data of the studies were conducted to determine the prevalence of migraine in Iran without a time limit until November 2022. This study was done following the statement of Preferred Reporting Items for Systematic Reviews and Meta-Analyses (PRISMA 2020) [13]. Therefore, after obtaining the documents, two reviewers (ND, HGH) independently and blind screened based on the title and abstract of the articles. After that, the full text of the articles was evaluated. If there is a difference in the approved articles, the opinion of the third reviewer (KV) was used as a criterion.

### Search strategy and inclusion and exclusion criteria

The inclusion criteria included all cross-sectional observational studies that investigated the prevalence of migraine, studies that: the full text of which was available and the information for the study, including the sample size and the criteria for diagnosing migraine; and studies that were published in English and Persian. The exclusion criteria included case report, case series, case-controls, and cohort studies.

After determining the inclusion and exclusion criteria, a systematic search of articles in three Iranian databases, MagIran and SID, with Iranian keywords and four international databases, Science Direct, Scopus, and ISI Web of Science and PubMed, with English keywords Done. The keywords used for searching in this study were selected according to the inclusion criteria based on published primary studies and Medical Subject Headings (MESH Terms) (in the reviewed database) and after a detailed examination of the questions of the study [14]. The search strategy was added to the supplementary appendix.

### Quality evaluation of the studies

The quality of confirmatory (observational) studies was measured in the previous stages using observational studies’ methodological quality measurement tool. The Strobe checklist [15] was used in this study. This checklist examines various aspects of writing a study, including the title, problem statement, study objectives, study type, statistical population, sampling method, determining the appropriate sample size, defining variables and procedures, study data collection tools, statistical analysis methods, and findings. The assessments in this checklist are done using 32 different items. The studies were given a score in the range of 0–32. Since this systematic reviewincluded studies with good or medium to high quality in the analysis, articles with a score of 16 and above were selected by the authors, and studies with a score of less than 16 were considered poor quality and were omitted.

### Data extraction and analysis

Pre-designed forms provided us with information. Various criteria were extracted and entered into the relevant forms, including the number of migraine patients and demographic information (first author, year of publication, country, continent, study population, and average age). We used Comprehensive Meta-Analysis software (Version 2) to analyze the data. The Begg and Mazumdar test was used at a significance level of 0.1. Also, this study used a corresponding Funnel plot and I2 test to check publication bias and heterogeneity, respectively. Also, subgroup analyses were performed based on gender and migraine diagnostic criteria.

## Results

**Fig. 1:**
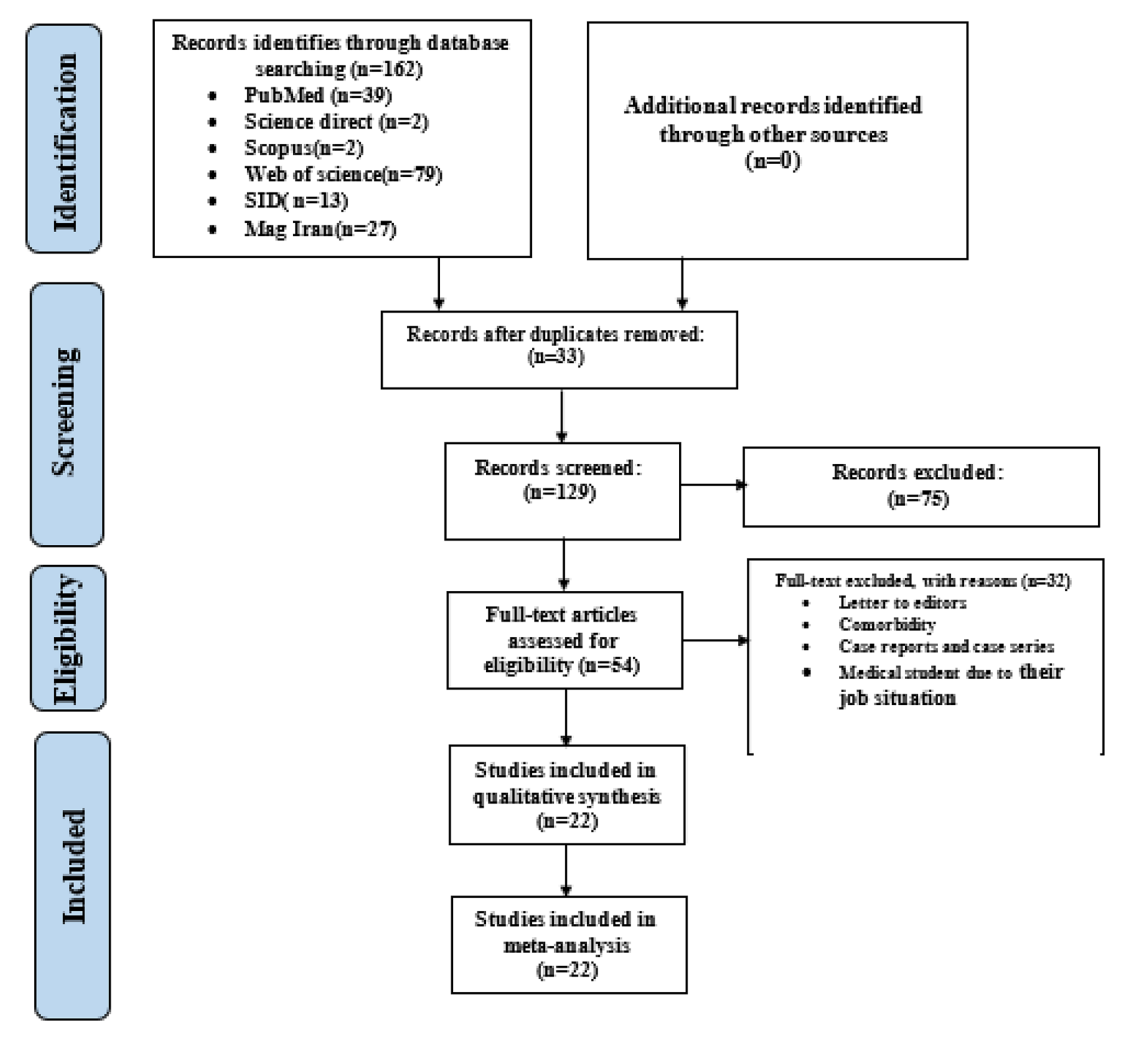
The flowchart indicating the steps involved in reviewing the studies included in the systematic review and meta-analysis (PRISMA 2020)

Based on the initial search in the following databases, 162 potential related articles were selected and transferred to the information management software (EndNote). Thirty-three studies were duplicated and excluded. In the screening phase, 75 articles were excluded by reading the title and abstract based on the inclusion and exclusion criteria from the remaining 129. In the eligibility evaluation stage, from the remaining 54 studies, 32 articles were excluded by reading the full text of the article based on the inclusion and exclusion criteria due to their irrelevance. Finally, 22 studies were included in the final analysis (Fig. 1).

### Prevalence in the population-based studies

**Fig. 2:**
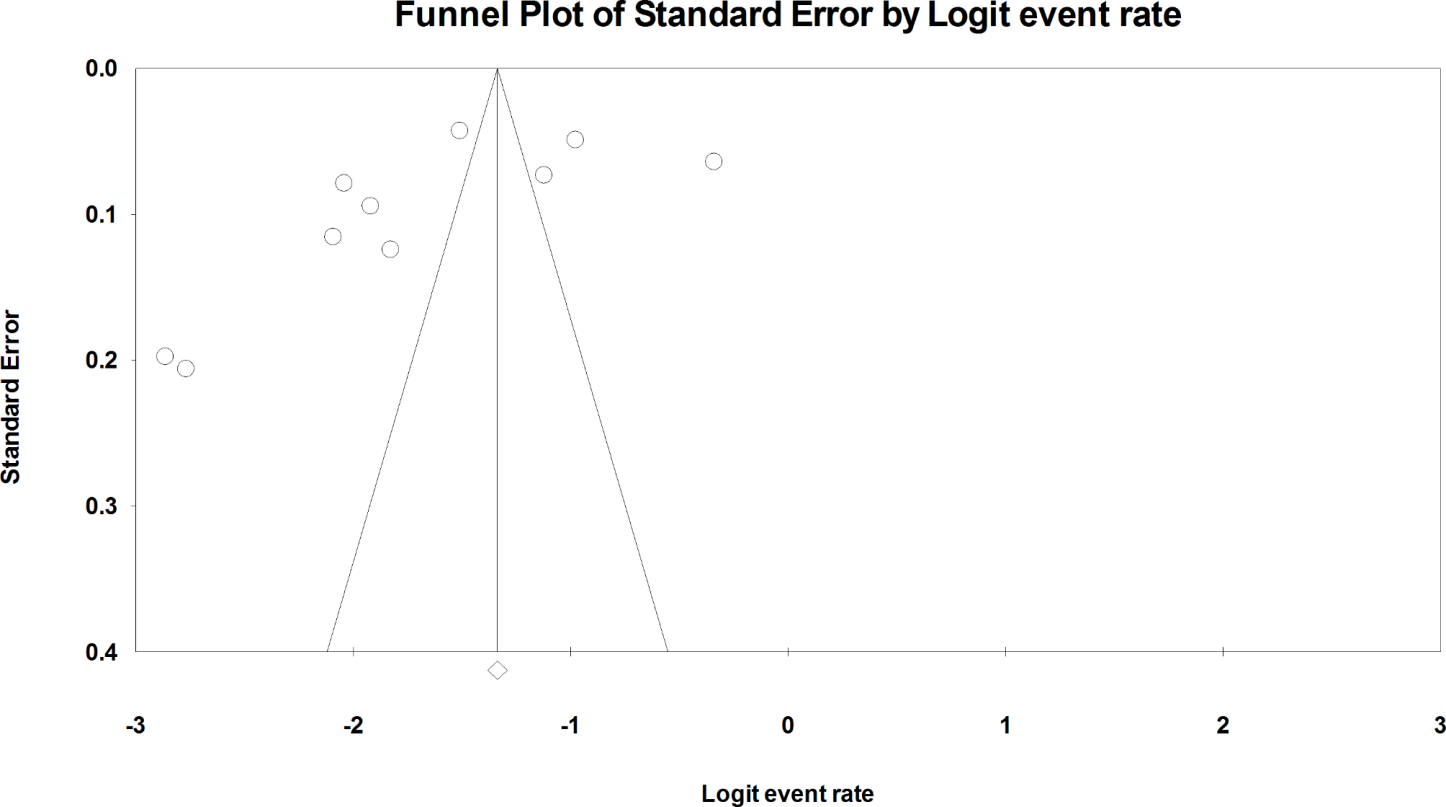
Funnel plot of migraine prevalence in the general population based on population-based studies

**Fig. 3:**
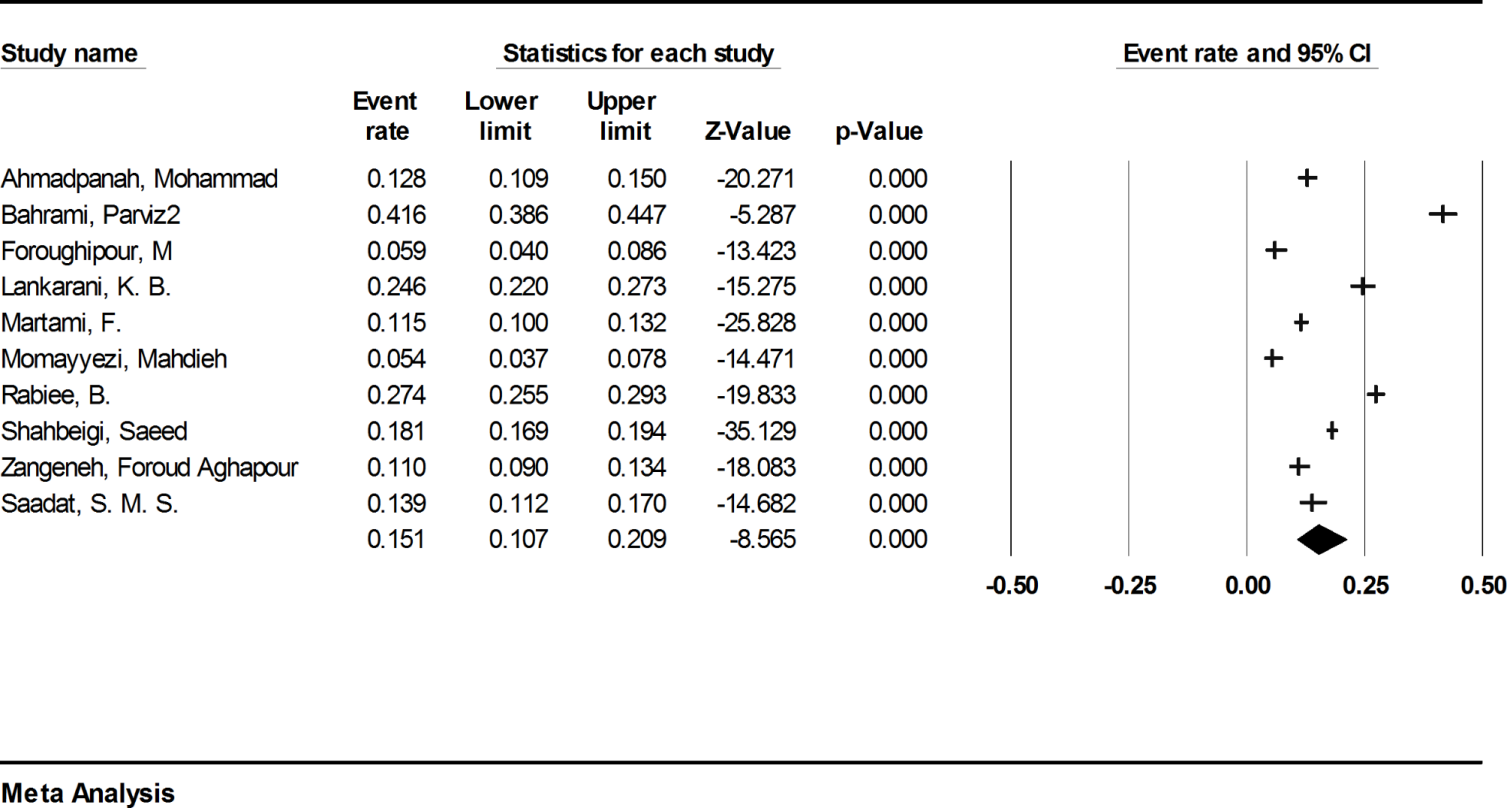
Forest plot of migraine prevalence in the general population based on population-based studies

The prevalence of migraine in the general population was calculated based on data from 10 studies (Table 1). These studies investigated 12,534 people in the age range of 10–95 years. Due to the high heterogeneity of the studies (I^2^ = 98.5%), statistical analysis was performed using the random effects model. The studies showed no publication bias among the studies (P = 0.107) Fig. 2. Based on this, the prevalence of migraine in the general population of Iran was calculated as 15.1% (CI 95%: 10.7–20.9) Fig. 3.

**Table 1:** Demographic information of population-based studies

first author		year	study type	setting	diagnostic criteria	mean age (SD) or range	Number of participants	Number of participants with migraine	male	Male with migraine	female	Female with migraine
Ahmadpanah, Mohammad [16]		2014	cross sectional	Hamedan	ICHD2	NR	1000	128	NR	NR	NR	NR
Bahrami, Parviz2 [8]		2012	cross sectional	Khorramabad	ICHD2	20–80	1000	416	NR	NR	NR	NR
Foroughipour, M [17]		2005	cross sectional	Mashhad	NR^1^	35	423	25	NR	NR	NR	NR
Lankarani, K. B. [18]		2017	cross-sectional	Kazerun	ICHD3	34.3	1001	246	383	54	618	192
Martami, F. [19]		2018	cross-sectional	Tehran	ICHD3	38.39(11.81)	1574	181	NR	NR	NR	NR
Momayyezi, Mahdieh [9]		2015	cross sectional	Yazd	ICHD2	25–75	500	27	NR	NR	NR	NR
Rabiee, B. [20]		2016	cross-sectional	Tehran	ICHD2	36.27(14.56)	2076	568	1264	271	1036	297
Shahbeigi, Saeed [21]		2013	cross sectional	Tehran	ICHD2	35.9(14.9)	3655	662	1206	127	2449	535
Zangeneh, Foroud Aghapour [22]		2012	cross sectional	Isfahan	ICHD2	31.3	764	84	NR	NR	NR	NR
Saadat, S. M. S. [23]		2014	Cross sectional	Rasht	ICHD2	11–75	541	75	NR	NR	NR	NR

SD: Standard deviation, ICHD: International Classification of Headache Disorders, NR: Not Reported,

^1^: diagnostic criteria are not mentioned in these studies

Based on Table 2, subgroup analyses were performed based on gender, diagnostic criteria, and migraine symptoms. Three studies were used to estimate the prevalence of migraine in men and women. The obtained results indicated that the prevalence of migraine in women is higher than in men (26.9% (CI 95%: 21.3–33.3) versus 14.9% (CI 95%: 8.9–23.7)).

**Table 2:** Analysis results of population-based studies

label	study	participants	Publication bias	I^2^	Analysis model	Prevalence%	CI 95%	P value
total	10	12,534	0.107	98.5%	Random model	15.1	10.7–20.9	P < 0.001
Male	3	2853	1	69.3%	Random model	14.9	8.9–23.7	P < 0.001
Female	3	4103	0.296	93.8%	Random model	26.9	21.3–33.3	P < 0.001
ICHD2	7	9536	0.132	98.6%	Random model	16.4	10.8–24.1	P < 0.001
ICHD3	2	2575	-	98.6%	Random model	17.1	7.7–33.6	0.001
Other criteria	1	423	-	99.2%	Fixed model	5.9	4-8.6	P < 0.001

CI: Confidence Interval, ICHD: International Classification of Headache Disorders

Migraine diagnostic criteria were also examined in this study. Based on this, in 7 studies, migraine diagnosis was made with the help of the second version of the International Classification of Headache Disorders (ICHD2). The prevalence of migraine based on diagnosis using the ICHD2 criteria was reported as 16.4% (CI 95%: 10.8–24.1) (Table 2). Two studies used ICHD3, and one used other diagnostic criteria. Migraine prevalence was calculated based on ICHD 3 criteria to be 17.1% (CI 95%:7.7–33.6((Table 1).

**Fig. 4:**
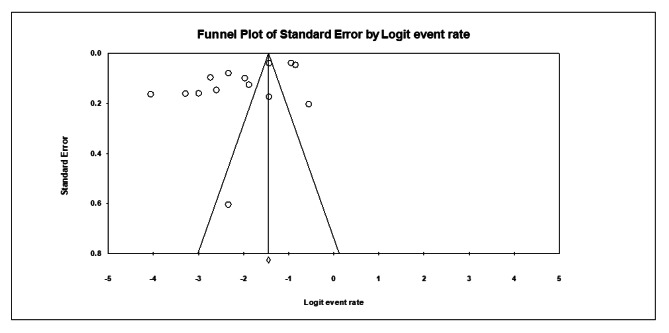
Funnel plot of migraine prevalence in schoolchildren

**Fig. 5:**
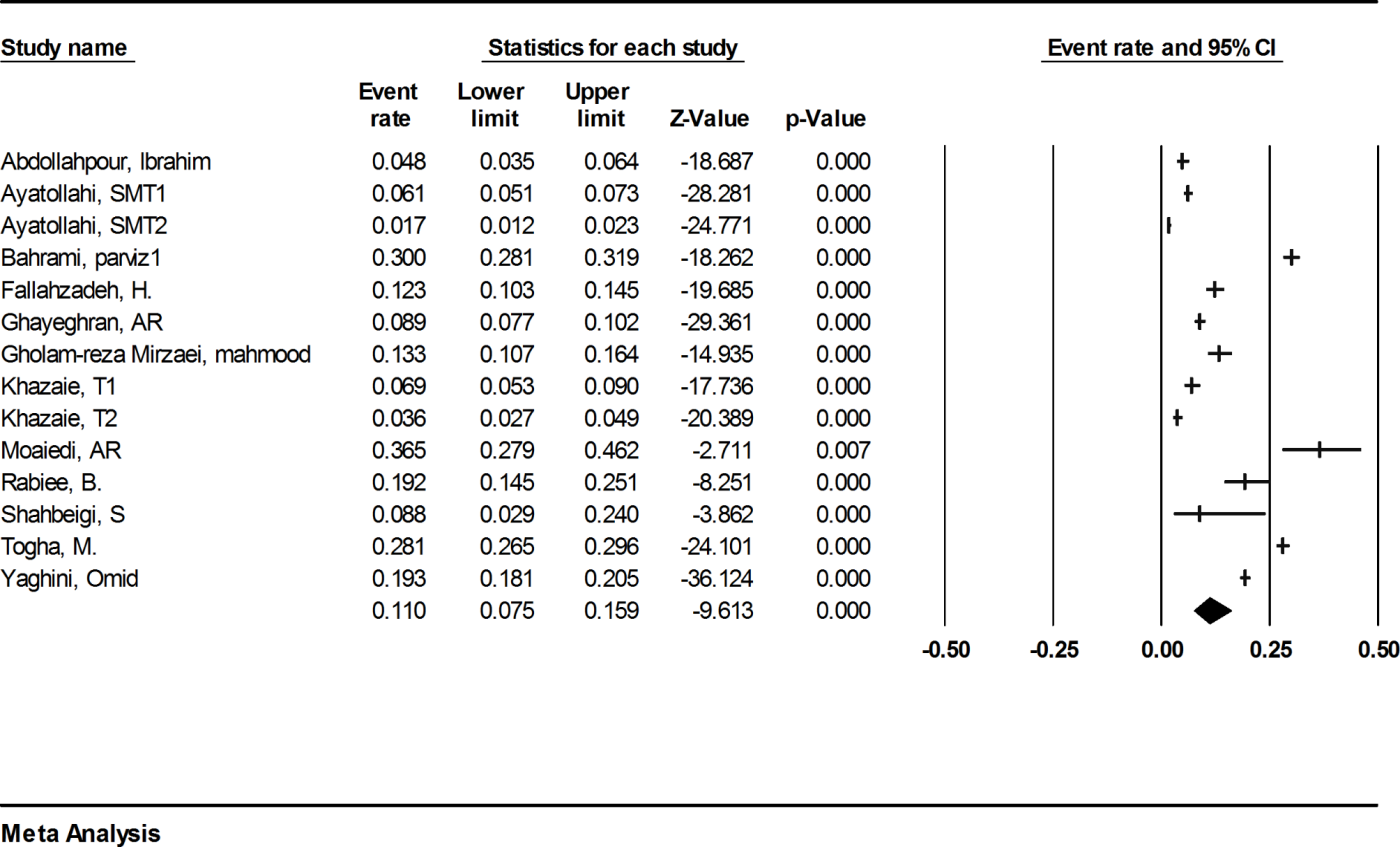
Forest plot of migraine prevalence in schoolchildren,

Prevalence of migraine in schoolchildren:

Fourteen studies examined schoolchildren (5–20 years). (Table 3). Due to the high heterogeneity among the studies, all statistical analyses were performed based on the random effects model (Table 4). The publication bias in the results of these studies was not significant (P = 0.443) Fig. 4. Based on the 14 studies (n = 20,130), the prevalence of migraine in schoolchildren was reported as 11% (CI 95%: 7.5–15.9%) (Fig. 5). The subgroup analyses showed the prevalence of migraine in girls was 8.6% (CI 95%: 5-14.2) and in boys 7.5% (CI 95%: 4.1–13.4). Statistical analysis by diagnostic criteria can also be seen in Table 4.

**Table 3:** Demographic information of studies in the age range of schoolchildren

first author	year	study type	setting	diagnostic criteria	range of age	mean age (SD)	Number of participants	Number of participants with migraine	Boy	Boy with migraine	Girl	Girl with migraine
Abdollahpour, Ibrahim [24]	2013	cross-sectional	Boukan	ICHD1	14–19	16.1(1.13)	857	41	389	22	468	19
Ayatollahi, SMT1 [25]	2002	cross sectional	Shiraz	ICHD1	11_18	NR	1868	114	NR	NR	1868	114
Ayatollahi, SMT2 [10]	2006	cross-sectional	Shiraz	ICHD1	6–13	NR	2226	38	1171	16	1055	22
Bahrami, parviz1 [26]	2006	cross-sectional	Khoramabad	NR^1^	NR	NR	2213	664	NR	NR	NR	NR
Fallahzadeh, H. [27]	2011	cross sectional	Yazd	ICHD1	12_15	13.3(0.98)	930	114	469	73	461	41
Ghayeghran, AR [28]	2004	cross sectional	Rasht	ICHD1	NR	15.37(1.11)	1965	174	1075	68	890	106
Gholam-reza Mirzaei, mahmood [29]	2004	cross sectional	Shahre kord	ICHD1	14–19	16.41(1.22)	550	73	NR	NR	550	73
Khazaie, T1 [30]	2011	cross sectional	Birjand	ICHD1	NR	15.81(1.02)	723	50	317	24	406	26
Khazaie, T2 [31]	2014	cross sectional	Birjand	ICHD2	7_11	9(1.41)	1107	40	556	21	551	19
Moaiedi, AR [11]	2004	Cross sectional	Bandar Abbas	ICHD2	5_16	9.72	104	38	NR	NR	NR	NR
Rabiee, B. [20]	2016	Cross sectional	Tehran	ICHD2	12–19	NR	213	41	NR	NR	NR	NR
Shahbeigi, S [21]	2013	Cross sectional	Tehran	ICHD2	10–15	NR	34	3	NR	NR	NR	NR
Togha, M. [32]	2022	cross sectional	13 provience in Iran	ICHD3	6_17	12.3(3.2)	3244	910	1531	419	1713	492
Yaghini, Omid [33]	2011	cross sectional	Isfahan	ICHD2	11_18	14.39	4096	791	2048	237	2048	554

SD: Standard deviation, ICHD: International Classification of Headache Disorders, NR: Not Reported,

1: diagnostic criteria are not mentioned in these studies

**Table 4:** Analysis results of studies among schoolchildren

label	study	participants	Publication bias	I^2^	Analysis model	Prevalence%	CI 95%	P value
schoolchildren (5–20 years)								
Total	14	20,130	0.443	98.9	Random	11	7.5–15.9	P < 0.001
Girl	10	10,010	0.107	98.7	Random	8.6	5-14.2	P < 0.001
Boy	8	7556	0.107	98.2	Random	7.5	4.1–13.4	P < 0.001
ICHD1	7	9119	0.133	96.3	Random	6.7	4.4–10	P < 0.001
ICHD2	5	5554	0.806	97.3	Random	14.5	6.9–27.8	P < 0.001
ICHD3	1	3244	-	-	-	28.1	26.5–29.6	P < 0.001
other	1	2213	-	-	-	30	28.1–31.9	P < 0.001
primary school (5–11 years)								
Total	4	4571	0.734	98.8	Random	5.2	1.3–18.7	P < 0.001
Girl	2	2264	-	99	Random	10.8	1.1–56.6	0.08
Boy	2	2087	-	98.9	Random	10.9	1.3–53	0.06
high school (12–20 years)								
Total	8	8820	1	99	Random	11.2	5.8–20.4	P < 0.001
Girl	6	4421	0.452	91.1	Random	8	5.6–11.2	P < 0.001
Boy	4	2250	1	92.3	Random	8.2	4.8–13.7	P < 0.001

CI: Confidence Interval, ICHD: International Classification of Headache Disorders

Four studies (n = 4571) were used to investigate the prevalence of migraine in primary schoolchildren. Based on the results obtained from these studies, the prevalence of migraine in primary schoolchildren was reported as 5.2% (CI 95: 1.3–18.7). Also, statistical analysis was done by gender, and the results were not statistically significant (Table 4). Also, the prevalence of migraine in high school children based on eight studies was 11.2% (CI 95%: 5.8–20.4). Also, the prevalence of migraine in boys was 8.2% (CI 95%: 4.8–13.7) and 8% (CI 95%: 5.6–11.2%) in girls. Table 4.

## Discussion

Based on the results of our study, the prevalence of migraine in the general population of Iran was 15.1% (CI 95%: 10.7–20.9). Subgroup analyses were performed based on gender, diagnostic criteria, and migraine symptoms. Three studies were used to estimate the prevalence of migraine in men and women. According to the results, the prevalence of migraine was reported in women at 26.9% (CI 95%: 21.3–33.3) and in men at 14.9% (CI 95%: 8.9–23.7). This result overlaps with the results of previous studies.

According to a study conducted in Germany, 23% of people over 20 have migraines [34]. A systematic review and meta-analysis conducted in 2014 examined 21 population-based studies. Based on these results, the statistical results of migraine in the general population of Africa have been reported as 5.61% (95% CI 4.61, 6.70;) which indicates less migraine compared to Iran, also the number of migraines in students has been reported as 14.89% (14.06, 15.74) [35]. In another systematic review aimed at the epidemiological investigation of migraine in Arab-speaking countries, 23 studies were examined. The prevalence of migraine in the general population was reported in the range between 2.6 and 32%. This study also observed that migraine in school children is in the range of 7.1–13.7% [36].

Different diagnostic criteria have been introduced to diagnose migraine worldwide. In 1988, the International Headache Society (IHS) introduced the first version of the International Classification of Headache Disorders (ICHD-1) [37]. Doctors and clinical researchers widely used this new diagnostic classification system. The ICHD-3 was released in early 2018. This version replaced the beta version of ICHD-3 released in 2013 [37]. Based on the results of our study, the diagnostic criteria of migraine were also examined in this study. Based on this, in 6 studies, migraine diagnosis was made with the help of the second version of the International Headache Criteria (ICHD2), and the prevalence of migraine based on the diagnosis using the ICHD2 tool was reported as 13.7% (CI 95%: 9.6–19.3). With the help of the ICHD3 tool, this value was reported as 17.1% (CI 95%: 7.7–33.6), and with the help of other diagnostic tools, this value was 17.6% (CI 95%: 1.9–69.7).

The prevalence of migraine is related to age and gender [38]. Out of this number, 77.3% of patients reported a positive family history of migraine. In that study, positive family history was defined as a migraine report in at least one relative, including father, mother, siblings, grandparents, and children [39].

Although migraine is a common disease in people aged,, its prevalence decreases in this group compared to adults [40]. According to the results, the prevalence of migraine in Iranian children and adolescents was 11% (CI 95%: 7.5–15.9%). Four studies were used to investigate the prevalence of migraine in children. These studies examined 4571 people aged 5–11 years. Based on the results obtained from these studies, the prevalence of migraine in children was reported as 5.2% (CI 95: 1.3–18.7). Also, a statistical analysis was done by gender, and the results obtained were not statistically significant.

A study stated that the prevalence of migraine in children increases with age [41]. According to another study, people aged 35 to 44 experienced minor migraines with the least intensity, and people over 45 experienced the most severe headaches [42]. In another study, it has been stated that the prevalence of migraine reaches its peak between the ages of 35 and 39 years, while the amount of this prevalence is lower at all ends of life (i.e., the relatively low prevalence in children or adolescents and the elderly) [38].

Although few studies have specifically addressed the prevalence of migraine in children, some population-based studies report a similar increase in migraine in women during puberty [43]. Based on the results of a Turkish study in school children (6–17 years), the prevalence of migraine was reported to be 26% [44], while a US study reported this rate to be 6% [45]. The ratio of migraine prevalence between men and women is not constant in all age ranges. Data from 40,892 men, women, and children in the 2003 US National Health Interview Survey (NHIS) showed that boys and girls have a similar prevalence of migraine until puberty, after which the prevalence increases in both sexes, but the increase of this value is more in women than in men [43].

Before puberty, girls and boys get migraines at about the same rate. It has been mentioned that migraine occurs earlier in boys [46]. The peak prevalence of migraine occurs in women of reproductive age, and women experience a more significant burden of migraine symptoms and disability than men. Sex hormones play an important role in migraine epidemiology [47]. The ratio of prevalence of migraine in women compared to men is approximately 3:1. Although the diagnostic criteria for migraine are the same for men and women, the clinical profile of migraine expression in women is more severe than in men [48]. Women with migraines experience more prolonged headaches, associated symptoms, migraine-related disabilities, a higher burden of co-morbidities, and worsening with age than men [43].

A review study examined related articles from China, Japan, and Korea. Based on the results of that study, the prevalence of migraine (based on IHS criteria) among adults was between 6 and 14.3%. Also, the highest complication prevalence was reported between 11 and 20% among women and between 3 and 8% among men [41]. Global prevalence data from the Global Burden of Disease Study in 2015 show that migraine is two to three times higher in women than in men, but in terms of prevalence, it peaks between 30 and 39 years of age in both sexes [43].

### Limitation

One of the limitations of this study was the small number of studies reviewed. Among the other limitations, we can point out the very high heterogeneity of studies, which made it impossible to perform meta-analysis with many studies. Also, since the studies examined a small number of people, the results of this study cannot be definitively generalized to the general population. Also, this study is not registered in PROSPERO.

## Conclusion

According to the results of this study, the prevalence of migraine in Iran’s general population was 15.1%, which is a significant amount. It was also observed that the prevalence of migraine in women is higher than in men. It was also observed that the prevalence of migraine in schoolchildren is 11%, which increases with age.

## Electronic supplementary material

Below is the link to the electronic supplementary material.

Supplementary Material 1

## Data Availability

Datasets are available through the corresponding author, upon reasonable request.
