# Supplementary material for: Prevalence of migraine in Iran: a systematic review and meta-analysis
Source: BMC Neurol. 2023 Apr 27;23:172. doi: 10.1186/s12883-023-03215-5 (PMC10134641; doi:10.1186/s12883-023-03215-5)
Supplement: Supplementary file 1 — Supplementary Material 1 [file 12883_2023_3215_MOESM1_ESM.pdf]

Appendix 1: search strategy

Database	Search strategy	Date	Number
PubMed	(prevalence[Title/Abstract] OR rate[Title/Abstract] OR outbreak[Title/Abstract] OR epidemiology[Title/Abstract] OR burden[Title/Abstract]) AND ("Migraine Disorder"[Title/Abstract] OR Migraine[Title/Abstract] OR "Migraine Headache"[Title/Abstract] OR "Acute Confusional Migraine"[Title/Abstract] OR "Hemicrania Migraine"[Title/Abstract] OR "Migraine Variant"[Title/Abstract] OR "Sick Headache"[Title/Abstract] OR "Abdominal Migraine"[Title/Abstract] OR "Cervical Migraine Syndrome"[Title/Abstract]) AND (iran[Title/Abstract] OR Islamic Republic of Iran[Title/Abstract])	2022.11.15	39
Science Direct	Title, abstract, keywords: (prevalence OR rate OR outbreak) AND (''Migraine Disorder'' OR Migraine OR "Migraine Headache" OR "Acute Confusional Migraine") AND (iran OR Islamic Republic of Iran)	2022.11.15	2
Scopus	TITLE-ABS-KEY(prevalence OR rate OR outbreak OR epidemiology OR burden) AND TITLE-ABS-KEY(''Migraine Disorder'' OR Migraine OR "Migraine Headache" OR "Acute Confusional Migraine" OR "Hemicrania Migraine" OR "Migraine Variant" OR "Sick Headache" OR "Abdominal Migraine" OR "Cervical Migraine Syndrome") AND TITLE-ABS-KEY(iran OR Islamic Republic of Iran)	2022.11.15	2
Web of science	TS=(prevalence OR rate OR outbreak OR epidemiology OR burden) AND TS=(''Migraine Disorder'' OR Migraine OR "Migraine Headache" OR "Acute Confusional Migraine" OR "Hemicrania Migraine" OR "Migraine Variant" OR "Sick Headache" OR "Abdominal Migraine" OR "Cervical Migraine Syndrome") AND TS=(iran OR Islamic Republic of Iran)	2022.11.15	79
SID *	Prevalence AND (migraine OR headache)	2022.11.15	13
Mag Iran*	Prevalence AND (migraine OR headache)	2022.11.15	27

*: Due to the fact that the supplementary file must be in English only, the English equivalent of the words is mentioned in the Iranian databases
